# Production of Superoxide Anions by Keratinocytes Initiates *P. acnes*-Induced Inflammation of the Skin

**DOI:** 10.1371/journal.ppat.1000527

**Published:** 2009-07-24

**Authors:** Philippe A. Grange, Christiane Chéreau, Joël Raingeaud, Carole Nicco, Bernard Weill, Nicolas Dupin, Frédéric Batteux

**Affiliations:** 1 Laboratoire de Recherche en Dermatologie, EA 1833, Faculté de Médecine, Université Paris Descartes, Paris, France; 2 Laboratoire d'Immunologie EA 1833, Faculté de Médecine, Université Paris Descartes, Paris, France; 3 ERTi «Plateforme d'étude du stress oxydant en oncologie et dans les maladies inflammatoires», Faculté de Médecine, Université Paris Descartes, Paris, France; 4 INSERM U749, Université Paris-sud, Faculté de Pharmacie, Chatenay-Malabry, France; 5 Service de Dermatologie-Vénéréologie, Hôpital Cochin – Pavillon Tarnier, AP-HP, Paris, France; Dartmouth Medical School, United States of America

## Abstract

Acne vulgaris is a chronic inflammatory disorder of the sebaceous follicles. *Propionibacterium acnes* (*P. acnes*), a gram-positive anareobic bacterium, plays a critical role in the development of these inflammatory lesions. This study aimed at determining whether reactive oxygen species (ROS) are produced by keratinocytes upon *P. acnes* infection, dissecting the mechanism of this production, and investigating how this phenomenon integrates in the general inflammatory response induced by *P. acnes*. In our hands, ROS, and especially superoxide anions (O_2_^•−^), were rapidly produced by keratinocytes upon stimulation by *P. acnes* surface proteins. In *P. acnes*-stimulated keratinocytes, O_2_^•−^ was produced by NAD(P)H oxidase through activation of the scavenger receptor CD36. O_2_^•−^ was dismuted by superoxide dismutase to form hydrogen peroxide which was further detoxified into water by the GSH/GPx system. In addition, *P. acnes*-induced O_2_^•−^ abrogated *P. acnes* growth and was involved in keratinocyte lysis through the combination of O_2_^•−^ with nitric oxide to form peroxynitrites. Finally, retinoic acid derivates, the most efficient anti-acneic drugs, prevent O_2_^•−^ production, IL-8 release and keratinocyte apoptosis, suggesting the relevance of this pathway in humans.

## Author Summary

Acne vulgaris is a chronic inflammatory disorder of the sebaceous follicles. It is the most common skin disease, affecting up to 80% of individuals at some point between the ages of 11 and 30 years. *Propionibacterium acnes* (*P. acnes*) plays a role in the development of inflammatory acne lesions, but whether it causes inflammation by itself or through indirect mechanisms is not clear yet. Therefore, by exposing epidermal cells to *P. acnes in vitro*, we tested whether reactive oxygen species (ROS) production (oxidative burst) was involved in the inflammatory process. We found that one particular ROS, superoxide anion, was generated by epidermal cells following *P. acnes* stimulation. This phenomenon is associated with the production of a soluble pro inflammatory molecule, IL-8, and epidermal cell death. The abrogation of *P. acnes*-induced oxidative burst by the most commonly used and most efficient treatments of acne suggests that superoxide anions produced by epidermal cells are critical in the development of acne inflammatory lesions.

## Introduction

Acne vulgaris is a chronic inflammatory disorder of the sebaceous follicles. Acne is the most common skin disease, estimated to affect up to 80% of individuals at some point between the ages of 11 and 30 years. Despite its common occurrence, the pathogenesis of acne is not fully understood. Excessive shedding of epithelial cells from the walls of follicles combined with increased amounts of sebum produced by associated sebaceous glands are two important factors that contribute to follicular obstruction. This obstruction leads to the formation of microcomedos, which are believed to precede lesions of acne. These microcomedos may evolve into clinically visible comedos and/or inflammatory lesions.

*Propionibacterium acnes* (*P. acnes*), a gram-positive anaerobic bacterium part of the normal skin flora, plays a critical role in the development of inflammatory lesions in acne [1]. Various mechanisms can explain the role of *P. acnes* in skin inflammation. First, it is widely accepted that inflammation may be induced by the immune response of the host to *P. acnes*. Chemotactic substances released from the bacteria attract polymorphonuclear leukocytes to the site of inflammation. Those cells are activated locally to produce inflammatory cytokines such as TNF-α, IL-1β, and IL-8 [2]. After phagocytosis of the bacteria, the attracted neutrophils are thought to release lysosomal enzymes and produce reactive oxygen species (ROS) that can damage the follicular epithelium.

Beside the immune response of the host, a direct effect of *P. acnes* on keratinocytes has also been suspected in the initiation of the inflammatory process. Indeed, *P. acnes* interacts with toll-like receptors TLR-2 and TLR-4 on keratinocytes [3]. This interaction induces the release of inflammatory cytokines such as IL-1α, IL-1β, IL-8, GM-CSF, and TNF-α [4],[5]. Although nothing is known about the interaction between *P. acnes* or any other bacteria with keratinocytes in terms of reactive oxygen species (ROS) production, purified tuberculine has been shown to activate TLR-2 on keratinocytes, leading to the production of ROS during tuberculosis infection [6]. In addition, *Vitreoscilla filiformis* has been identified to activate MnSOD as an inducible free-radical scavenger in keratinocytes [7]. Furthermore, keratinocytes are known to produce ROS upon exposure to toxic compounds such as inorganic arsenic [8] or to ultraviolet radiations [9],[10]. Whatever the mechanism implicated in the induction of skin inflammation by *P. acnes*, ROS are probably involved in that process since the production of hydrogen peroxide (H_2_O_2_) is increased in neutrophils from acne patients [11]. Moreover, the decrease in superoxide dismutase (SOD) activity in patients with acne lesions [12] is correlated with the severity of acne [13].

ROS are short-lived small molecular structures that are continuously generated at low levels during the course of normal aerobic metabolism. They are also part of the inflammatory process that aims at killing or eliminating invasive microorganisms and/or eliminating damaged tissular structures. Among the large number of ROS that have been described, superoxide anion (O_2_^•−^) and hydrogen peroxyde (H_2_O_2_) play prominent roles. On the other hand, the interaction between O_2_^•−^ and nitric oxide (NO), leads to the formation of highly reactive peroxynitrites (ONOO^•−^). ROS interact strongly with a variety of molecules including lipids, proteins, and nucleic acids. Produced in large amounts, ROS can lead to apoptotic or necrotic cell death. To counteract the overproduction of ROS, skin is equipped with antioxidant mechanisms including anti-oxidant enzymes such as superoxide dismutase (SOD) that detoxifies O_2_^•−^, catalase, and glutathione peroxidase (GpX) that uses reduced glutathione (GSH) to detoxify H_2_O_2_ into water [14].

In this work, we have used an *in vitro* model to investigate ROS production by keratinocytes upon *P. acnes* stimulation. We have dissected the control mechanisms of this production, and investigated how they fit into the general inflammatory response induced by *P. acnes*.

## Results

### ROS production by *P. acnes*-stimulated keratinocytes is dose-and time-dependent

**Figure 1:**
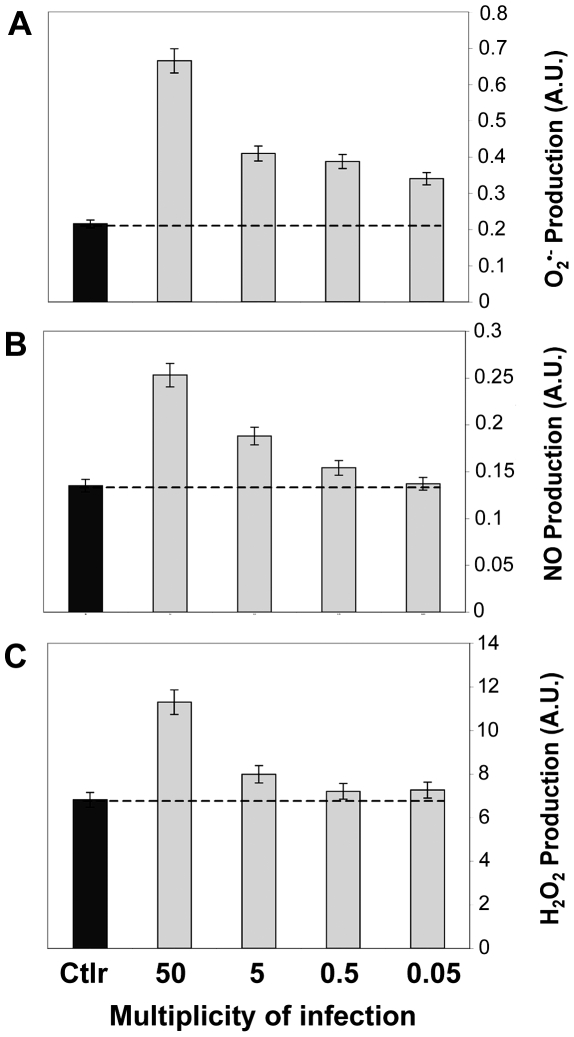
ROS production by *P. acnes*-stimulated keratinocytes. HaCaT cells were incubated for 18 h with *P. acnes* at an MOI of 50, 5, 0.5, and 0.05 (gray bars). Control experiments were done on HaCaT cells alone (black bars). Measurement of (A) superoxide anion, (B) nitric oxide, and (C) hydrogen peroxide was realized by spectrofluorometry as described in Materials and Methods. Data are means±SD of two separate experiments.

**Figure 2:**
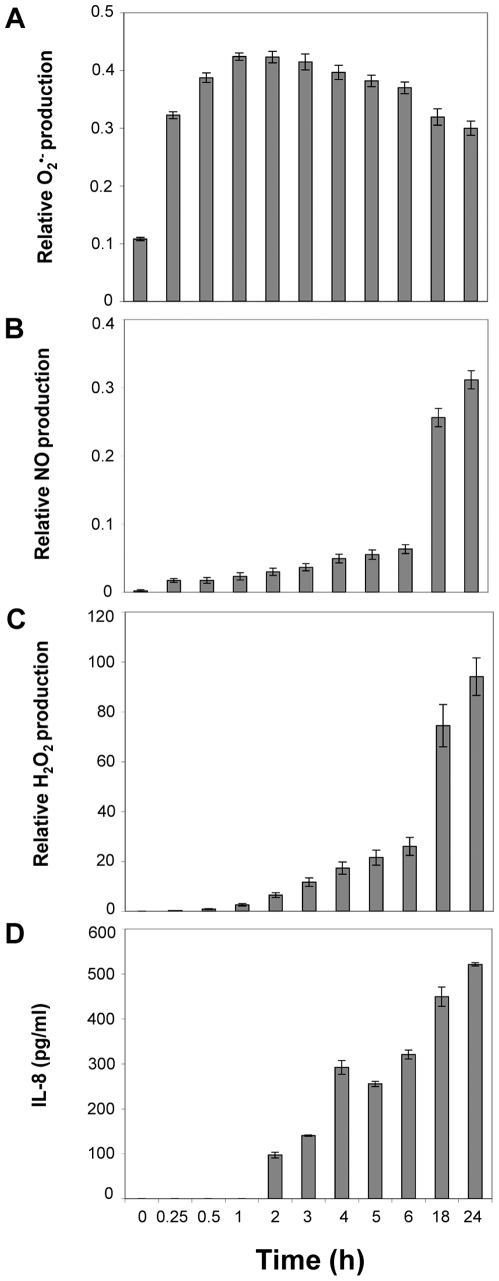
Kinetics of ROS versus IL-8 production in *P. acnes*-stimulated keratinocytes. HaCaT cells were incubated with *P. acnes* (MOI of 50) for a period of time ranging from 0.25 to 24 h. (A) Superoxide anion, (B) nitric oxide, (C) hydrogen peroxide levels were determined by spectrofluorometry as described in Materials and Methods. (D) IL-8 production was determined by ELISA as described in Materials and Methods. Data are means±SD of two separate experiments.

**Figure 3:**
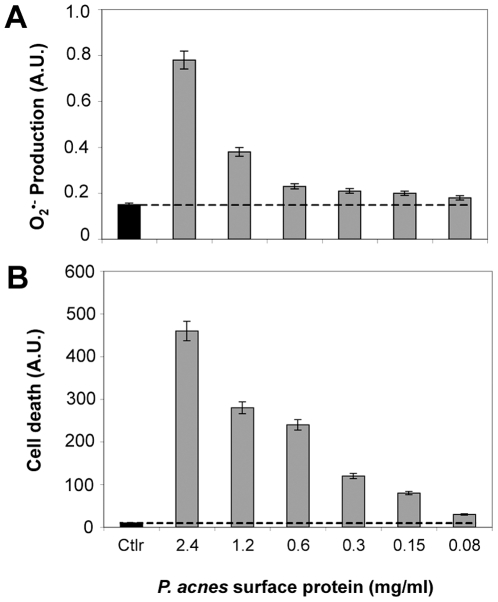
Superoxide anion production by keratinocytes stimulated with *P. acnes* total surface proteins extract. HaCaT cells were incubated for 18 h in the presence of several *P. acnes* surface proteins whose concentrations ranged from 0.08 to 2.4 mg/ml (gray bars). Control experiments were done on HaCaT cells in presence of PBS (gray bars). Measurements of (A) superoxide anion, and (B) cell death were performed by spectrofluorometry with DHE and YO-PRO-1, respectively as described in Materials and Methods. The amount of cells present in wells after 18 h of incubation was 66, 73, 77, 85, 89, and 98%, respectively. Data are means±SD of two separate experiments.

*P. acnes* increased the production of O_2_^•−^, NO and H_2_O_2_ by the immortalized keratinocyte cell line HaCaT in a dose-dependent manner (Figure 1A, B and C). At the highest concentration of *P. acnes*, O_2_^•−^, NO and H_2_O_2_ levels were increased by 85% (P<0.05), 44.5% (P<0.05) and 41% (P<0.05), respectively. We then evaluated the kinetics of ROS production (Figure 2). The production of O_2_^•−^, was significantly increased 15 min after *P. acnes* stimulation (P<0.05). The production reached its peak one hour after the stimulation, then progressively declined (Figure 2A). In contrast, both NO and H_2_O_2_ productions increased slowly and reached their highest levels after 24 h of incubation with *P. acnes* (Figures 2B and C). Since keratinocytes stimulated by *P. acnes* can produce IL-8 [3], we next compared the kinetics of ROS production with that of IL-8 production upon *P. acnes* stimulation (Figure 2D). Significant levels of IL-8 protein appeared 2 h after incubation with *P. acnes* (P<0.05) and increased along with ROS production. Altogether, these results indicate that the production of ROS, and especially of O_2_^•−^, is a very early event occurring almost immediately after the stimulation of keratinocytes with *P. acnes*. The production of O_2_^•−^ by HaCat keratinocytes was identical whether the cells had been stimulated by an extract of *P. acnes* surface proteins or by the whole bacteria. O_2_^•−^ production was measured using DHE, and cell death estimated using YO-PRO-1 were dose-dependent (Figure 3).

### Origin of ROS produced by keratinocytes stimulated with *P. acnes*

**Figure 4:**
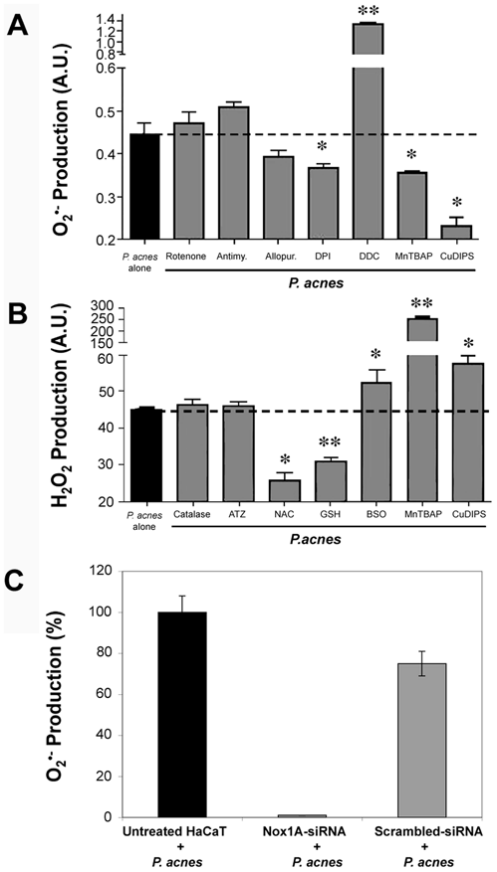
ROS detoxification pathways involved in *P. acnes*-stimulated keratinocytes. (A) Superoxide anions and (B) hydrogen peroxide productions by HaCaT cells were determined after incubation for 18 h with *P. acnes* (MOI of 50) alone (black bars) or with *P. acnes* in the presence of specific modulators of enzymatic systems involved in ROS metabolism (gray bars). The concentrations used were the following: 40 µM rotenone, 40 µM antimycin, 40 µM allopurinol, 40 µM DPI, 2 mM DDC, 400 µM ATZ, 0.8 mM BSO, 100 µM manganese [III] tetrakis (5,10,15,20)-benzoic acid porphyrin (MnTBAP), 400 µM copper[II]diiosopropylsalicylate (CuDIPS), 3.2 mM N-acetylcysteine (NAC), 1.6 mM GSH, 20 U catalase. (C) HaCaT cells were pretreated with the Nox1A-siRNA sequence or with a scrambled sequence as described in Materials and Methods. Superoxide anions production was measured after stimulation by *P. acnes* (MOI of 50) in untreated HaCaT (black bar) or in siRNA pretreated HaCaT (gray bars). ROS production was measured by spectrofluorometry as described in Materials and Methods. Data are means±DS of two separate experiments.

Superoxide anions can originate from the mitochondrial complex I or III of the respiratory chain, or from the cytosolic enzymes NAD(P)H oxidase or xanthine oxidase. Incubation of *P. acnes*-stimulated keratinocytes with rotenone and antimycin that inhibit the mitochondrial respiratory chain complexes I and III, respectively, did not significantly alter the production of O_2_^•−^ (Figure 4A). Incubation of *P. acnes*-stimulated keratinocytes with DPI (a NAD(P)H oxidase inhibitor) significantly decreased O_2_^•−^ production (P<0.03), while incubation with allopurinol (a xanthine oxidase inhibitor) had no effect (Figure 4A). To confirm that Nox is the main source of O_2_^•−^ in keratinocytes stimulated by *P. acnes*, the level of Nox1 was knocked down using RNA interference. The small interfering RNA (siRNA) Nox1A-siRNA was used as described previously [15]. Nox1A-siRNA dramatically decreased the production of O_2_^•−^ upon stimultion by *P. acnes* in transfected-keratinocytes, with nearly 100% inhibition after 3 h of stimulation (Figure 4C). Keratinocytes treated with scrambled sequence siRNA produced similar levels of O_2_^•−^ as non-transfected cells. These results demonstrated that O_2_^•−^ is mainly produced by NAD(P)H oxidase in *P. acnes*-stimulated keratinocytes.

### ROS detoxification pathways involved in *P. acnes*-stimulated keratinocytes

In order to determine the pathways implicated in the detoxification of ROS produced by *P. acnes*-stimulated keratinocytes, we used specific modulators of the enzymatic systems involved in ROS metabolism. Superoxide anions are converted into hydrogen peroxide by SOD. Inhibiting SOD by the specific inhibitor DDC significantly increased O_2_^•−^ production by *P. acnes*-stimulated keratinocytes (P<0.003) (Figure 4A). By contrast, incubation of keratinocytes with MnTBAP or CuDIPS, two SOD mimics, significantly decreased O_2_^•−^ production by *P. acnes*-stimulated keratinocytes (P<0.05 and P<0.04, respectively) (Figure 4A). Hydrogen peroxide is converted into H_2_O by two sets of enzymes, catalase and the GSH/GPx system. The elevation of hydrogen peroxide levels can be caused either by an increase in superoxide dismutation as observed following incubation with MnTBAP (P<0.004) or CuDIPS (P<0.02) or by a decrease in the detoxification pathways (Figure 4B). Specific inhibition of catalase by aminotriazol (ATZ) or addition of exogenous catalase, had no effect on the levels of hydrogen peroxide (Figure 4B). Thus, the catalase pathway is not involved in the control of hydrogen peroxide detoxification in our system. Depleting GSH with BSO, inhibited GPx and significantly increased H_2_O_2_ production (P<0.05), while adding exogenous GSH or its precursor NAC, significantly decreased H_2_O_2_ levels (P<0.004 and P<0.01, respectively) (Figure 4B). Those results highlight the role of GSH/GPx in keratinocytes to counteract the overproduction of ROS induced by *P. acnes*: superoxide anions are dismuted by SOD into hydrogen peroxide, which is further detoxified into H_2_O through the GSH/GPx pathway.

### ROS toxicity and production of nitrosyl residues by *P. acnes*-stimulated keratinocytes

**Figure 5:**
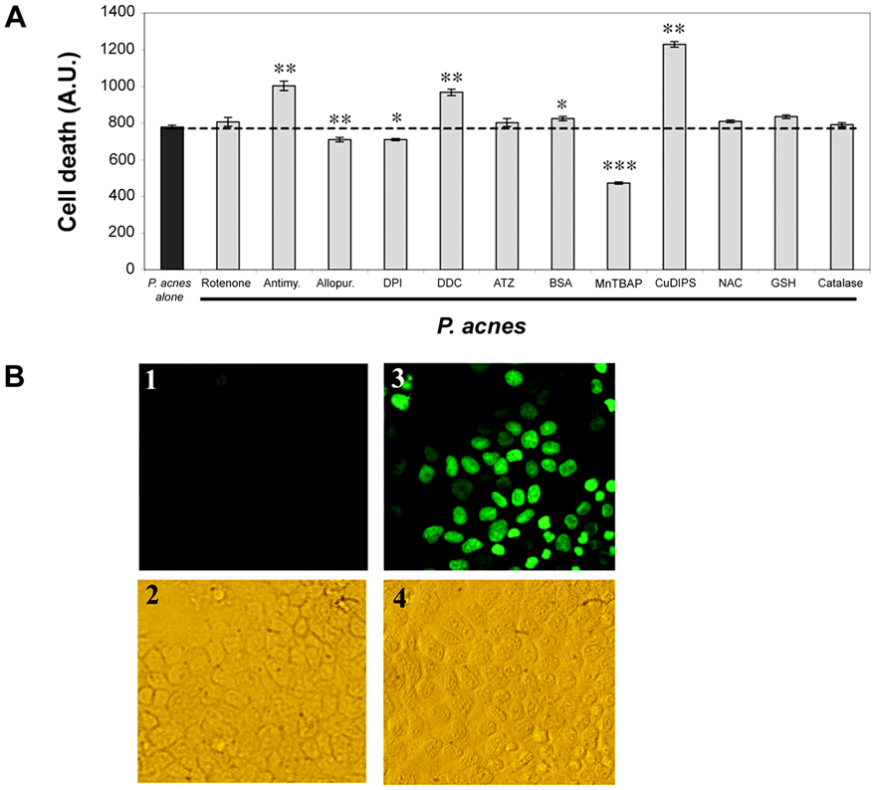
Relationship between *P. acnes*-induced O_2_^•−^ production and keratinocyte apoptosis. (A) HaCaT cells were incubated for 18 h with *P. acnes* (MOI of 50) (black bars) or with *P. acnes* in the presence of modulators of ROS production (gray bars). The concentrations used were 40 µM rotenone, 40 µM antimycin, 40 µM allopurinol, 40 µM DPI, 2 mM DDC, 400 µM ATZ, 0.8 mM BSO, 100 µM manganese[III]tetrakis(5,10,15,20)-benzoic acid porphyrin (MnTBAP), 400 µM copper[II]diiosopropylsalicylate (CuDIPS), 3.2 mM N-acetylcysteine (NAC), 1.6 mM GSH, 20 U catalase. Cell death was assessed spectrofluorometrically as described in Materials and Methods and expressed as means±SD from two experiments carried out in duplicates. (B) Induction of DNA fragmentation in HaCaT cells following incubation with *P. acnes* was assessed by TUNEL staining as described in Materials and Methods. Panels 1 and 2 correspond to HaCaT cells alone. Panels 3 and 4 correspond to HaCaT cells incubated with *P. acnes* for 18 h at an MOI of 50.

Given the high toxicity of ROS, we were prompted to investigate if the levels of O_2_^•−^ produced by keratinocytes could impact cellular viability. The apoptosis of keratinocytes induced by *P. acnes* alone was estimated by YO-PRO-1 (Figure 5A) and TUNEL staining (Figure 5B). In order to determine the nature of ROS involved in *P. acnes*-induced cellular toxicity, we pre-treated keratinocytes wih specific modulators of the enzymes involved in the production of O_2_^•−^ and H_2_O_2_ and measured the death of keratinocytes upon stimulation with *P. acnes*. Inhibition of superoxide anions by allopurinol, DPI or MnTBAP, significantly decreased *P. acnes*-induced keratinocyte apoptosis (P<0.005, P<0.005, P<0.001, respectively), whereas DDC and antimycin, two compounds that increase O_2_^•−^ production, increased cell death (P<0.007 and P<0.01, respectively) (Figure 5A). CuDIPS, a mimic of the cytosolic superoxide dismutase, increased cell death. This is explained by the cytotoxic properties of this molecule on the cells. No major effect was observed on the rate of cell death with molecules modulating H_2_O_2_ production, such as ATZ, BSO, NAC, GSH or catalase.

**Figure 6:**
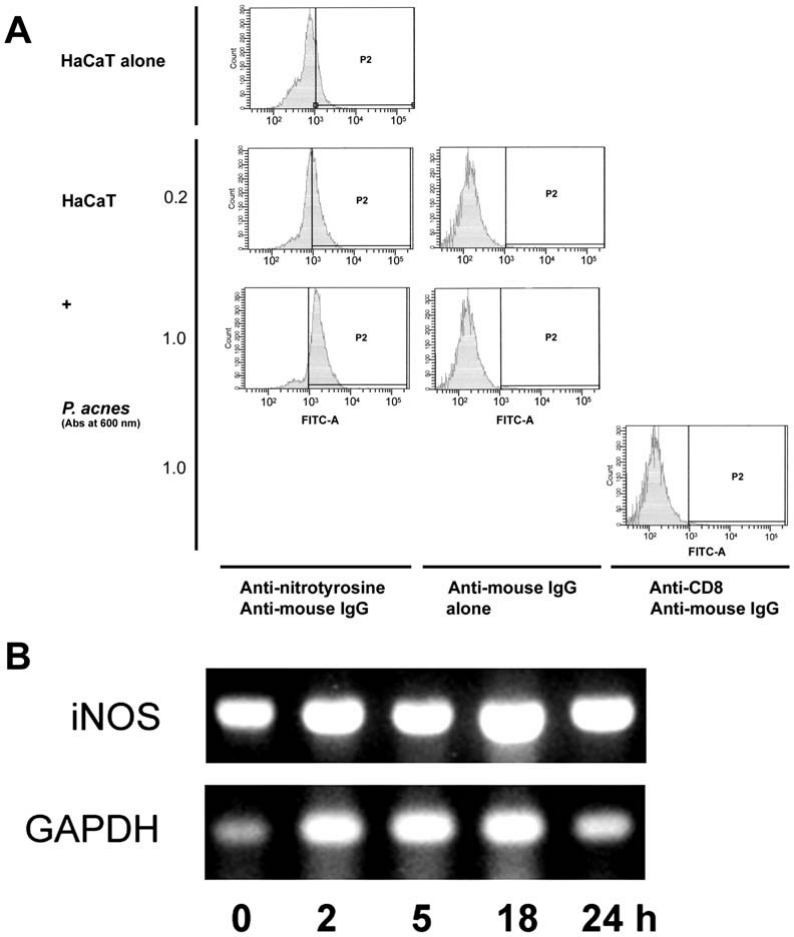
Nitrosyl residue formation and iNOS expression in *P. acnes*-stimulated keratinocytes. HaCaT cells were incubated for 18 h with *P. acnes* (A_600 nm_ = 0.2 and 1.0) and harvested following trypsine treatment after removal of bacteria. (A) Cells were incubated with primary mouse monoclonal antibody to nitrotyrosine or with anti-CD8 monoclonal antibody as control. Bound antibodies were detected using a goat anti-mouse IgG-FITC secondary antibody. Cells were then analyzed by flow cytometry as described in Materials and Methods. (B) Total RNA was extracted and the iNOS and GAPDH mRNA expression was analysed by RT-PCR. PCR fragments were visualized under U.V. on a 1.7% agarose gel after staining with ethidium bromide (1 µg/ml).

Peroxinitrites result from the combination of O_2_^•−^ and NO. They are highly reactive metabolites that create nitrosyl residues on proteins and alter their functions. Therefore, the levels of nitrosyl residues not only reflect the intensity of the oxidative attack but are also markers of the cellular damages created by the oxidative burst. As shown by flow cytometry, 3-nitrosotyrosyl residues were dose-dependently increased from with very low concentrations of *P. acnes* (Figure 6A). This result is in agreement with the observation that the nitric oxide synthase (NOS) is activated in keratinocytes stimulated with *P. acnes*. The expression of iNOS was steady in keratinocytes during a period of time of 24 h as determined by RT-PCR (Figure 6B) and by RT-qPCR (data not shown). This data is consistent with those presented in Figure 1, showing that NO was produced early after *P. acnes* incubation, making its interaction between O_2_^•−^ and NO possible. Altogether, those experiments suggested that the toxicity of O_2_^•−^ produced by keratinocytes stimulated with *P. acnes* was dependent on the combination with NO and the production of nitrosyl residues.

### *P. acnes*-induced O_2_^•−^ production controls IL-8 levels

**Figure 7:**
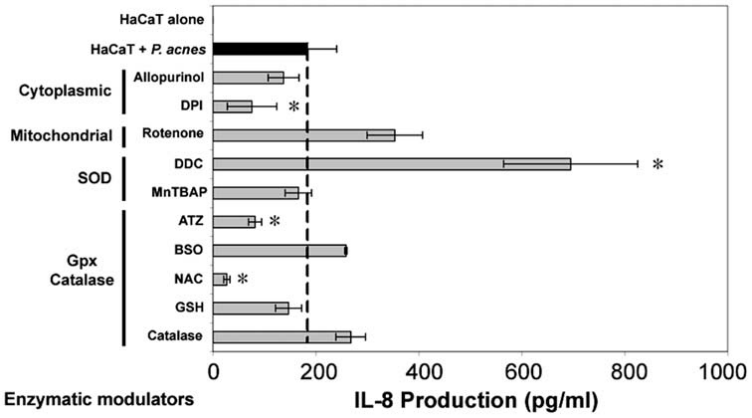
Relationship between ROS and IL-8 productions in *P. acnes*-stimulated keratinocytes. HaCaT cells were incubated for 18 h with *P. acnes* alone (MOI of 50) (black bar) or with *P. acnes* in the presence of ROS modulators (gray bars). The concentrations used were 40 µM rotenone, 40 µM antimycin, 40 µM allopurinol, 40 µM DPI, 2 mM DDC, 400 µM ATZ, 0.8 mM BSO, 100 µM manganese[III]tetrakis(5,10,15,20)-benzoic acid porphyrin (MnTBAP), 400 µM copper[II]diiosopropylsalicylate (CuDIPS), 3.2 mM N-acetylcysteine (NAC), 1.6 mM GSH, 20 U catalase. IL-8 concentration was measured in culture supernatants by ELISA as described in Materials and Methods. Data are means±SD of two separate experiments.

In order to evaluate the role of O_2_^•−^ in the production of IL-8 by *P. acnes*-stimulated keratinocytes, we measured the levels of IL-8 produced in presence of the various ROS modulators (Figure 7). All the molecules that inhibited O_2_^•−^ production also decreased IL-8 synthesis, but only the decrease induced by DPI reached statistical significance (P<0.03). If all the molecules that increased O_2_^•−^ levels also increased IL-8 production, only the massive increase caused by DDC reached significance (P<0.04) (Figure 7). Both ATZ and NAC significantly decreased IL-8 production. Since it has previously been shown that ATZ has no effect on H_2_O_2_ production while NAC and GSH do (Figure 4), these results suggest that the effects of ATZ and NAC on IL-8 production are independent of the regulation of H_2_O_2_ and are more likely linked to intrinsic properties of those products. Altogether these results suggest that the toxicity of ROS on *P. acnes*-stimulated keratinocytes is mainly caused by O_2_^•−^ which also exerts a positive effect on IL-8 production.

### Role of the scavenger receptor CD36 in the production of superoxyde anions

**Figure 8:**
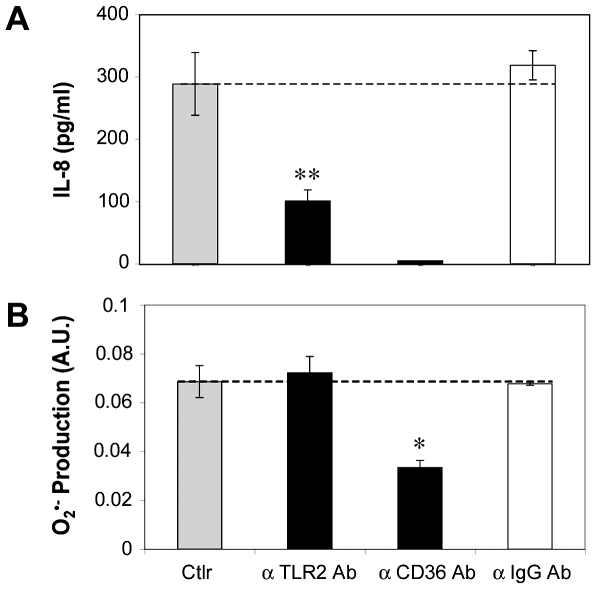
*P. acnes* induces the production of superoxide anions via CD36. HaCaT cells were pretreated 2 h with human anti-TLR-2 or human anti-CD36 monoclonal antibodies (black bars), goat anti-IgG antibody (white bar) and incubated 3 h with *P. acnes* (A_600 nm_ = 1.0). Control experiments were run in parallel with *P. acnes* alone (gray bar). (A) IL-8 concentrations were measured in culture supernatants by ELISA, as described in Materials and Methods and are showed minus the value obtained with the HaCaT cells treated with the mAb alone. (B) O_2_^•−^ production was measured spectrofluometrically over a period of 3 h as described in Materials and Methods. Data are presented as means±SD of three separate experiments.

Since O_2_^•−^ elicits IL-8 production by keratinocytes stimulated with *P. acnes*, we investigated which surface proteins could be implicated in the recognition of *P. acnes*. *P. acnes*-stimulated keratinocytes were incubated with antibodies to TLR-2 or CD36, and IL-8 and O_2_^•−^ productions measured (Figure 8). The antibody directed to TLR-2 was known as a blocking agent for the production of IL-8 by keratinocytes after stimulation by *P. acnes* [3]. This was confirmed by the reduction in IL-8 production by 65% (P = 0.01) (Figure 8A), whereas no change was observed in O_2_^•−^ production (Figure 8B). However, when *P. acnes*-stimulated keratinocytes were incubated in the presence of the antibody to CD36, the production of O_2_^•−^ was reduced by 51% (P = 0.03) and the production of IL-8 was completely abolished.

### Effect of ROS on *P. acnes* growth

**Figure 9:**
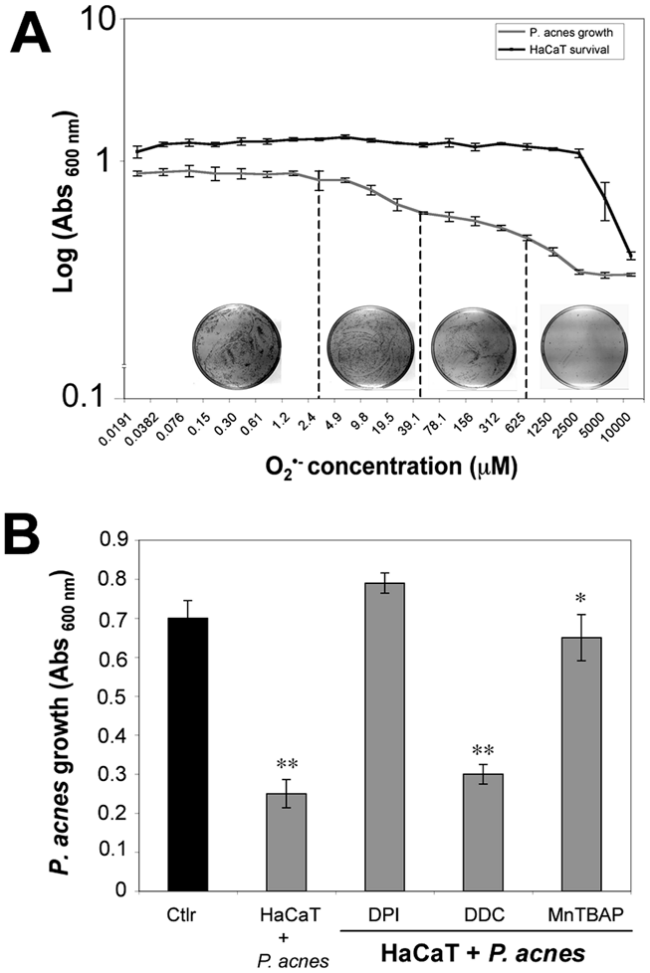
Effect of ROS on *P. acnes* growth. (A) HaCaT cells (dark line) and *P. acnes* suspension (Abs_600 nm_ = 0.5) (gray line) were incubated for 2 h under appropriate conditions with various concentrations of chemically generated O_2_^•−^ ranging from 0,0191 µM to 10 mM. The viability of HaCaT cells was determined by the MTT assay as described in Materials and Methods. *P. acnes* suspension was then incubated for 5 days at 37°C under anaerobic conditions and the bacterial growth was evaluated by culturing bacteria on RCM solid media and by measuring the Abs at 600 nm. (B) HaCaT cells were preincubated with 40 µM DPI, 2 mM DDC, 50 µM MnTBAP, and stimulated with *P. acnes* (MOI of 50) in DMEM 10% SVF without antibiotics at 37°C, 5% CO_2_. After 5 h of stimulation, liquid RCM was added to each well and the incubation for 5 days at 37°C under anaerobic conditions was started. *P. acnes* growth was then evaluated by measuring the Abs at 600 nm and by culturing bacteria on RCM solid media. Control experiment was run in parallel with the *P. acnes* in DMEM 10% SVF without antibiotics alone.

We first compared the relative sensitivity of HaCaT cells and *P. acnes* to the toxic effect of O_2_^•−^. HaCaT cells and *P. acnes* were incubated separately with a solution containing O_2_^•−^. The growth of *P. acnes* was dose dependently inhibited by O_2_^•−^ while the HaCaT cells appear to be more resistant than *P. acnes* at the same O_2_^•−^ concentration (Figure 9A). We then tested the hypothesis that the ROS produced by keratinocytes, and particularly O_2_^•−^, could be responsible for the inhibition of the growth of *P. acnes* (Figure 9B). When *P. acnes*-stimulated keratinocytes were preincubated with MnTBAP, a MnSOD mimic that detoxifies O_2_^•−^, or with DPI that inhibits NAD(P)H oxydase, the growth of the bacteria was restored. Reciprocally, when keratinocytes where preincubated with DDC, a SOD inhibitor, the bacterial growth was decreased.

### Anti-acne drugs inhibit O_2_^•−^ production, IL-8 synthesis and keratinocyte apoptosis

**Figure 10:**
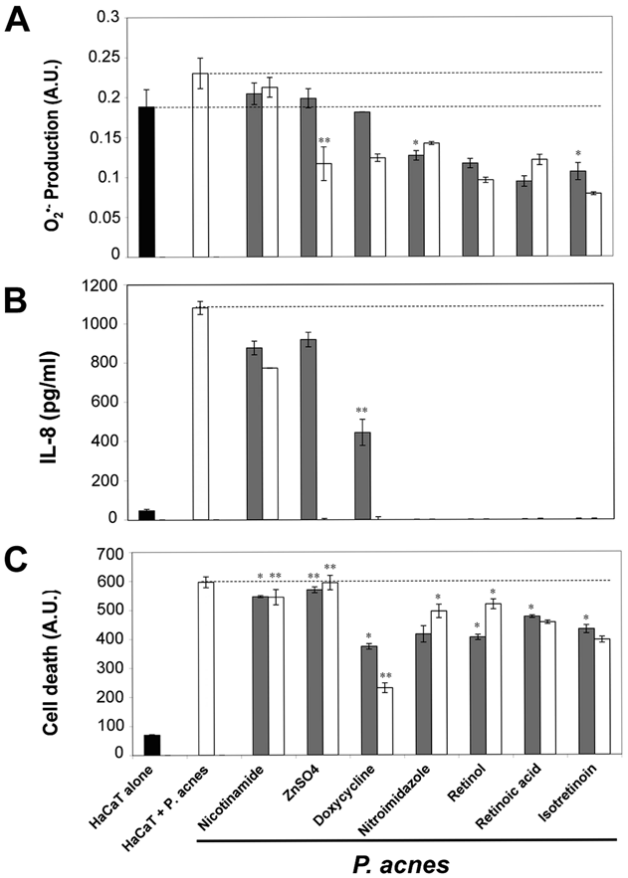
Effect of anti-acne treatments on O_2_^•−^ and IL-8 production in *P. acnes*-stimulated keratinocytes. HaCaT cells were untreated (black bar) or incubated for 18 h with *P. acnes* (MOI of 50) alone (white bar) or with *P. acnes* in the presence of nicotinamide, zinc sulfate, doxyciclyne, nitroimidazole, retinol, retinoic acid, and isotretinoin at 0.01% (gray bars) or 0.05% (white bars). (A) O_2_^•−^ production was determined spectrofluorometrically with DHE, (B) IL-8 concentration was measured by ELISA, (C) and cell death was assessed spectrofluorometrically using YO-PRO-1 as described in Materials and Methods. Data are means±SD of two separate experiments.

In order to evaluate the effects of the most common drugs used in the treatment of acne, HaCaT cells were stimulated by *P. acnes* in the presence of ZnSO_4_, doxycycline, nicotinamide, nitroimidazol, retinol, retinoic acid, or isotretinoin (Figure 10). The production of superoxide anions was reduced by all the drugs tested, at least at the highest concentration (0.05%), except for nicotinamide (Figure 10A). IL-8 production was reduced neither by ZnSO_4_ at low concentration (0.01%) nor by nicotinamide, but all the others drugs tested were effective. This is particularly the case for retinoic acid derivates that completely abolished IL-8 production (Figure 10B). The percentage of cells present in the wells after incubation ranged from 68 to 91% (Figure S1). All the drugs except ZnSO_4_ and nicotinamide reduced the apoptosis of keratinocytes stimulated by *P. acnes* at least at the highest concentration tested (0.05%). This is particularly the case for retinoic acid derivates (P<0.03 in all cases) and for the antibiotics doxycycline (P<0.02) and nitroimidazole (P<0.03) (Figure 10C). The rate of cell death in the presence of the various compounds alone ranged from 0 to 26% (Figure S2). Altogether, these results suggest that the anti-acne drugs are active on the production of O_2_^•−^ and IL-8 as well as on the decrease in the death rate of keratinocytes.

## Discussion

This report describes the production of ROS by keratinocytes upon bacterial infection by *P. acnes*. The production of superoxide anions takes place at least one hour prior to that of nitrix oxide and hydrogen peroxide. The same kinetics is observed following UV radiation or arsenite intoxication [8].

Superoxide anions can originate from the cytosolic enzymes NAD(P)H oxidase, or xanthine oxidase, or from the complexes I or III of the mitochondrial respiratory chain. The use of DPI, an inhibitor of NAD(P)H oxidase and more specifically knocking down Nox1 by small RNA interference clearly shows that, in *P. acnes*-stimulated keratinocytes, O_2_^•−^ is produced by NAD(P)H oxidase. This data is in line with a recent report showing that NAD(P)H oxidase is the major source of UVA-induced ROS in human keratinocytes where mitochondria are rapidly damaged after UVB exposure [16]. However, to date, no link between a specific damage of the mitochondrial respiratory chain and the production of O_2_^•−^ has been established [15]. Under our experimental conditions, superoxide anions are dismuted by superoxide dismutase to form H_2_O_2_, which is further detoxified into water by the GSH/GPx system and not by the catalase pathway. In contrast, H_2_O_2_ generated by UVB applied to keratinocytes is detoxified through both the catalase and the GPx pathways. Usually, catalase finely tunes down H_2_O_2_ levels, while the glutathione system (GPx and reduced glutathione) is more specialized in buffering acute oxidative stress. This is probably what happens in the case of *P. acnes* infection.

However, the key-element for *P. acnes*-induced apoptosis of keratinocytes is O_2_^•−^ and not H_2_O_2_. O_2_^•−^ can be toxic *per se* or following its combination with NO to form peroxynitrites (ONOO^•−^), a phenomenon that requires the activation of inducible nitric oxide synthase (iNOS). We confirm that *P. acnes* induces the formation of nitrotyrosine residues on proteins, a footprint of *in vivo* peroxinitrite production [17]. Similarly, keratinocytes exposed to UVB or arsenite produce both O_2_^•−^ and NO, potentially leading to peroxinitrite formation [8],[18]. In our model, the production of NO by *P. acnes*-stimulated keratinocytes is correlated with the steady expression of iNOS, as already observed in keratinocytes [18]. Those data suggest that the cytotoxicity mediated by ROS in our model involves the overproduction of O_2_^•−^ and also the nitrosylation of amino acid residues on proteins.

**Figure 11:**
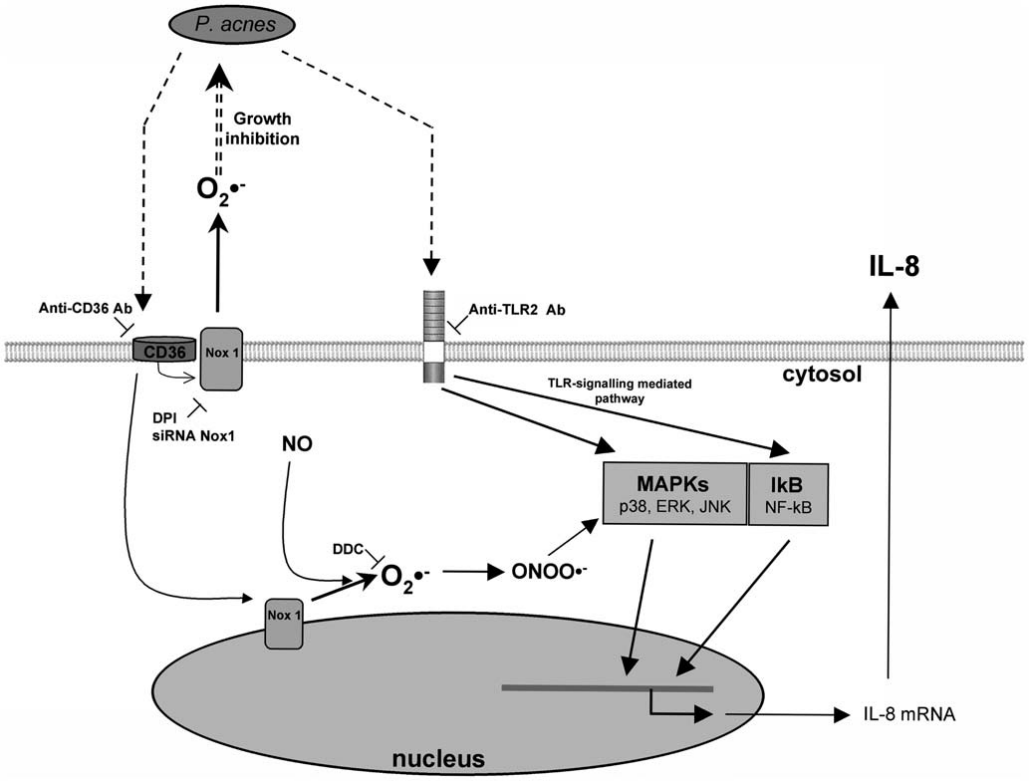
Proposed molecular mechanisms through which *P. acnes* induces ROS and IL-8 production in keratinocytes. Surface components of *P. acnes* are recognized by both CD36 and TLR-2. CD36 triggers the production of O_2_^•−^ through the NADPH oxidase pathway (NOX) and combines with NO to form peroxinitrites which, in turn, activate p38 and ERK MAPKs, thus contributing to IL-8 production. In parallel, IL-8 production is activated through the TLR-2 signalling pathway.

Keratinocytes are the first line of defense against external aggressions; they participate in the innate immune response by secreting soluble factors with chemotactic activity for leukocytes and neutrophils. Thus, *P. acnes* triggers the secretion of IL-1α, TNF-α [4], and the chemokine IL-8 [3] which have been implicated in the inflammatory process of acne. Using activators and inhibitors of the O_2_^•−^ production, we have been able to modulate the production of IL-8 upon stimulation by *P. acnes*. Particularly, DPI an inhibitor of the NADPH oxidase, significantly decreases IL-8 production, whereas DDC, a SOD inhibitor that increases O_2_^•−^ levels, dramatically increases IL-8 production by keratinocytes. The question was then to determine the pathway through which *P.acnes* stimulates keratinocytes. Several previous observations suggested the implication of the Toll-like receptor (TLR) pathway. TLRs can recognize conserved molecular structures at the surface of bacteria. TLR-2, present at the surface of keratinocytes [19],[20], is upregulated in acne lesions [21] and is potentially involved in the recognition of *P. acnes* during the inflammatory process [22]. Moreover, *P. acnes*-stimulated TLR-2 induces IL-8 release by keratinocytes [3],[23]. We have observed a time-lag between the early production of O_2_^•−^ and the secretion of IL-8 that occurs 2 h later, that probably corresponds to the activation of the TLR-signaling mediated pathway. Therefore, we hypothesized that the molecular mechanism responsible for O_2_^•−^ production is TLR-independent. Indeed, whereas blocking TLR-2 with a monoclonal antibody decreases the production of IL-8 as described previously [3], it has no effect on O_2_^•−^ production. We also tested the role of CD36, a scavenger molecule expressed on keratinocytes [24]. The generation of ROS by the NAD(P)H oxidase-NOX system has already been observed following the activation of scavenger receptors *in vitro* [25] and *in vivo* in a murine model of cerebral ischemia [26]. This receptor is a sensor of microbial diacylglycerides that signals via the TLR-2/6 heterodimer. In response to bacterial lipoteichoic acid (LTA) and diacylated lipoproteins, CD36 associates with TLR-2/6 [24],[27]. Although it does not express LTA, *P. acnes* expresses a closely related amphiphilic antigen, a lipoglycan containing mannosyl, glucosyl, galactosyl residues, and an amino sugar, diaminohexuronic acid [28],[29]. We observed that, blocking CD36 with a monoclonal anti-CD36 antibody in *P. acnes*-stimulated keratinocytes, significantly decreases both the level of O_2_^•−^ and that of IL-8. In our model, IL-8 secretion is triggered by the binding of *P. acnes* to TLR-2 and modulated by the generation of superoxide anions resulting from the binding of *P. acnes* to CD36. In phagocytic cells, Nox1 oxidizes NADPH on the cytosolic side of the cellular membrane and reduces oxygen across the membrane to generate O_2_^•−^ which contributes to the killing of *P. acnes* [30]. On the other hand, in keratinocytes, Nox1 is localized in the nucleus [31] and could release O_2_^•−^ into the cytoplasm. Therefore, we hypothesized that nuclear Nox1 could generate O_2_^•−^ which combine with steadily NO to form peroxinitrites. Peroxinitrites activate p38 and ERK in the MAPK pathways, contributing to the tight regulation of IL-8 production by O_2_^•−^ [32],[33] (Figure 11). In addition, O_2_^•−^ produced by keratinocytes upon stimulation with *P. acnes*, counteract the growth of the bacteria. Those results highlight a new mechanisms by which keratinocytes participate in the innate immune response to pathogens.

Finally, the inhibition of O_2_^•−^ production, IL-8 release and keratinocyte apoptosis by retinoic acid derivates, the most efficient anti-acneic drugs, demonstrates the relevance of these pathways *in vivo*. In addition, our data are in agreement with the observations that , retinoic acid can induce MnSOD mRNA in a human neuroblastoma cell line and decrease TPA-induced O_2_^•−^ production in mouse keratinocytes [34]. In conclusion, keratinocytes are not mere targets of the innate immune response but are directly involved in the defence mechanisms aiming at eliminating pathogens. In response to *P. acnes*, keratinocytes can produce massive amounts of ROS that, in return, inhibit bacterial growth. Those ROS do not only eliminate the bacteria but also generate inflammation. Thus, we hypothesize that the severity of acne depends on the balance between the ability of the *P. acnes* strain to induce a potent immune response [3] and the capability of the host to generate and to detoxify the ROS produced [11],[13]. Therefore, inhibiting this inflammatory reaction using appropriate antioxidant molecules could be considered as a potential treatment of acne.

## Materials and Methods

### Bacterial culture

*P. acnes* strain 6919 was obtained from the American Type Culture Collection (Manassas, VA) and grown under anaerobic conditions in reinforced clostridial liquid and solid medium (RCM) (Difco Laboratories, Detroit, MI) at 37°C during 5 days in order to reach stationary phase. Typically, 100 ml of RCM were used and bacteria were harvested after centrifugation at 7,000 *g* for 10 min at 4°C. Pellets were pooled and washed in about 30 ml of cold PBS and centrifuged again as described above. Finally, the bacterial pellet was suspended in PBS or DMEM. From this suspension, dilutions of 10^5^ to 10^8^ CFU/ml were prepared, resulting in a multiplicity of infection (MOI) of 0.05 to 50 bacteria per cell in 0.1 ml of inoculum. To obtain total surface protein extract, the bacteria were scraped in the presence of 2 ml of PBS [1.5 mM KH_2_PO_4_, 2.7 mM Na_2_HPO_4_.7H_2_O, 0.15 M NaCl (pH 7.4)] from the solid RCM. The bacterial suspension was heated at 60°C for 20 min and the bacteria removed by centrifugation at 16,000 g for 20 min at 4°C. The supernatant containing total surface proteins was subjected to ammonium sulfate precipitation at 60% of saturation for 1 h under stirring. The precipitated proteins were recovered after centrifugation at 22,000 g for 30 min at 4°C, then resuspended in PBS, and extensively dialyzed against PBS. Protein concentration was determined by the method of Lowry using BSA as standard described by Peterson [35].

### Cell culture and stimulation

The human keratinocyte cell line HaCaT was grown in Dulbecco's modified Eagle's medium-Glutamax-I (DMEM) (Invitrogen, Cergy Pontoise, France) supplemented with 10% heat-inactivated fetal calf serum (Invitrogen), 20 mM L-glutamine, 1 mM sodium pyruvate, and antibiotic/antimycotic solution (10 U/ml Pencillin, 10 µg/ml Streptomycin, 0.25 µg/ml Amphoterin) (Invitrogen) at 37°C in humidified atmosphere containing 5% CO_2_ as described [36]. The cell line was routinely tested to assess the absence of *Mycoplasma* infection. For stimulation experiments, HaCaT cells were incubated with the *P. acnes* suspension adjusted at the appropriate concentration in buffer solution for the desired period of time at 37°C in 5% CO_2_.

### Measurement of ROS production by spectrofluorimetric analysis

HaCaT cells (2.10^4^/well) were seeded in 96-well plates (Corning Costar, Brumath, France). After 18 h, cells were washed three times in PBS and incubated with 100 µl per wells of 5 µM DHE (for determination of O_2_^•−^) or 5 µM H_2_-DCFDA (for determination of H_2_O_2_) or 5 µM DAF_2_-DA (for determination of NO) for 30 min as described previously [37],[38],[39]. Fluorescent probes were purchased from Molecular Probes (Eugene, OR, USA). After three washes, cells were incubated with 100 µl of a suspension of *P. acnes* in PBS (Abs at 600 nm = 0.5) and fluorescence intensity was recorded every hour over a period of 5 h. Fluorescence excitation/emission maxima were for DAF_2_-DA: 495/515 nm, for DHE: 480/610 nm and for H_2_-DCFDA: 507/525 nm. At the end of the experiment, the number of adherent cells was evaluated by the crystal violet assay as described below. O_2_^•−^, NO and of H_2_O_2_ were assayed by spectrofluorimetry on a Fusion spectrofluorimeter (PackardBell, Paris, France). Levels of ROS were calculated in each sample as follows: reactive oxygen species rate (arbitrary units/min/10^6^ cells) = (fluorescence intensity [arbitrary units] at T5h – fluorescence intensity [arbitrary units] at To/300 minutes/number of adherent cells as measured by the crystal violet assay, and were expressed as arbitrary unit (A.U.).

### Origin and modulation of *P. acnes*-induced superoxide anions

HaCaT cells (2.10^4^/well) were seeded in 96-well plates and incubated for 18 h in complete medium alone or with the following molecules: 2 mM diethyldithiocarbamate (SOD inhibitor), or 400 µM CuDIPS (Cu/Zn SOD mimic), or 100 µM MnTBAP (MnSOD mimic), or 40 µM rotenone (inhibitor of mitochondrial complex I) or 40 µM antimycine (inhibitor of mitochondrial complex III), 40 µM diphenyliodonium (inhibitor of NADPH oxidase), or with 40 µM allopurinol (inhibitor of xanthine oxidase). Cells were then washed three times in PBS and incubated with 100 µl per well of 5 µM DHE for 30 min. After three washes, cells were incubated with 100 µl of a suspension of *P. acnes* (Abs at 600 nm = 0.5) and fluorescence intensity was recorded every hour over a period of 5 h as previously described. At the end of the experiment, the number of adherent cells was evaluated by the crystal violet assay. The levels of O_2_^•−^ were calculated as described above.

### Origin and modulation of *P. acnes*-induced hydrogen peroxide in HaCaT cells

HaCaT cells (2.10^4^/well) were seeded in 96-well plates and incubated for 18 hours in complete medium alone or with the following molecules: 3200 µM reduced glutathione, 800 µM N-acetylcysteine, or 400 µM CuDIPS, or 100 µM MnTBAP, or 100 µM D,L-buthionine-[S,R]-sulfoximine (inhibitor of glutathione reductase), or 400 µM aminotriazol (inhibitor of catalase), or 20 U PEG-catalase (cell permeable catalase). Cells were then washed three times in PBS and incubated with 100 µl per wells of 5 µM H_2_-DCFDA for 30 minutes. After three washes, cells were incubated with 100 µl of a suspension of *P. acnes* (Abs at 600 nm = 0.5) and fluorescence intensity, was read at a fluorescence excitation wavelength of 507 nm and at an emission wavelength of 525 nm, and was recorded every hour over a period of 5 hours. At the end of the experiment, the number of adherent cells was evaluated by the crystal violet assay. The levels of H_2_O_2_ were calculated in each sample as described above.

### Silencing Nox1 by RNA interference

Nox1 silencing was performed as previously described [15]. We used the Nox1-A siRNA primer with the sequence sense 5′-ACAAUAGCCUUGAUUCUCAUGGUAA-3′, anti-sense 5′-UUACCAUGAGAAUCAAGGCUAUUGU-3′, located at 750 bp. A scrambled siRNA duplex as negative control was used with the sequence sense 5′-ACACCGAAGUUUCUUGUACGUAUAA-3′, anti-sense 5′-UUAUACGUACAAGAAACUUCGGUGU-3′ (MWG Biotech, Les Ulis, France). At 24 h before transfection, HaCaT cells were transferred onto 96-well plates at the density of 1.10^4^ cells/well and transfected with 10 nM of each siRNA duplex using INTERFERin™ transfection reagent (Polyplus transfection, Illkirch, France) for 4 h in serum free DMEM without antibiotics. Then, complete DMEM medium was added and the cells were incubated for 48 h. Western blot using specific antibody against Nox1 (Santa Cruz Biotechnology Inc., Santa Cruz, CA) was used to assess the reduction of Nox1 protein production as previously described [15]. The level of Nox1 using Nox1A-siRNA was decreased by 86%, whereas scrambled siRNA did not affect the Nox1 level (Figure S3).

### Measurement of cell death by spectrofluorimetric analysis

Cell death was estimated spectrofluorometrically using the fluorescent probe YO-PRO-1 (Molecular Probes) on a Fusion spectrofluorimeter (Packard Bell). HaCaT cells (2.10^4^/well) were seeded in 96-well plates and incubated for 18 h in complete medium alone or with the following molecules: 2 mM diethyldithiocarbamate, or 40 µM rotenone, or 40 µM antimycine, 40 µM diphenyliodonium, or with 40 µM allopurinol, or 1600 µM reduced glutathione, 3200 µM N-acetylcysteine or 400 µM CuDIPS, or 100 µM MnTBAP, or 800 µM D,L-Buthionine-[S,R]-sulfoximine, or 400 µM aminotriazol, or 20 U PEG-catalase. Cells were then washed three times in PBS and incubated with 100 µl per well of a suspension of *P. acnes* (Abs at 600 nm = 0.5) for 24 h in complete medium. After three washes in PBS, cells were incubated with 10 µM YO-PRO-1 for 30 min. Cell death was measured by reading at an excitation wavelength of 480 nm and an emission wavelength of 525 nm. The level of cell death was estimated in each sample by the fluorescence intensity [arbitrary units] reflecting the disruption of the cell membranes.

### Measurement of cell death by TUNEL staining

HaCaT cells incubated or not with *P. acnes* were fixed in 3.7% buffered formaldehyde directly onto the 96-well plate. Cells were then subjected to TUNEL assay using the TACS™ TdT-Fluorescein *In situ* apoptosis detection kit (R&D Systems Inc., Minneapolis, MN) following the manufacturer's intructions. Briefly, after fixation, cells were permeabilized by Proteinase K and incubated with the reaction mixture containing Terminal deoxynucleotidyl Transferase (TdT) and biotinylated-conjugated dNTPs for 1 h at 37°C. After washing, biotinylated nucleotides were detected by incubating cells with a streptavidin-fluorescein conjugate for 20 min at room temperature in the dark. After removing the excess of fluorescein conjugate by washing in 0.1% Tween 20 in PBS, labeled DNA was examined under a fluorescence microscope.

### Cell viability assays

Crystal violet staining was used to determine the number of adherent cells in 96-well plates. Briefly, after incubation with the test compound, the culture medium was discarded and the cells were incubated with a 0.05% crystal violet solution (Sigma) for 30 min at room temperature. After washing with PBS, 100% methanol was added, and the absorbance was measured spectrophotometrically at 540 nm on an ELISA multiwell reader.

The MTT (1-(4,5-dimethylthiazol-2-yl)-3,5-diphenylformazan) assay was performed to test cell viability in 96-well plates. The cells were incubated with a 0.2% MTT solution in cell culture medium for 4 h at 37°C. The MTT solution was then discarded and DMSO added to solubilize the MTT-formazan cristals produced in living cells. After thorough mixing, the absorbance was measured at 540 nm.

### Measurement of nitrosyl residues by flow cytometry

HaCaT cells were incubated in presence of two *P. acnes* concentrations (Abs at 600 nm = 0.2 and 1.0) for 18 h at 37°C. Cells were washed twice with cold PBS, harvested after trypsinization and fixed with 3.5% paraformaldehyde in PBS for 15 min at 4°C. After washing in PBS, cells were permeabilized in 1% NP-40 and incubated with FITC-labelled anti 3-nitrotyrosine monoclonal antibody (Clone 1A6, Upstate Cell Signalling Solutions, Lake Placid, NY, USA) at 6.4 µg/ml for 1 h at 4°C. After three washes, cells were pelleted and suspended in 1 ml of PBS, then analyzed by flow cytometry (FACScalibur, Becton Dickinson, Mountain View, CA). Control experiments were perfomed by incubating the cells with a FITC-labelled irrelevant IgG of the same isotype under the same conditions as described above.

### ELISA

Human IL-8 protein concentration was measured in the supernatants of HaCaT cells using the Quantikine® human IL-8 immunoassay kit (R&D Systems Inc., Mineapolis, MN) according to the manufacturer's instructions. We used serial dilutions of recombinant human IL-8 for standard curve. The optical density was determined at 450 nm at a wavelength correction of 540 nm.

### RNA isolation and RT-PCR

Total RNA was isolated with TRIzol® reagent (Invitrogen) according to the manufacturer's instructions and treated with DNAse I (Roche Molecular Biochemical). RNA concentration was determined by reading the absorbance at 260 nm. Complementary DNA (cDNA) was generated from 2 µg total RNA using the oligo(dT) primer (MWG Biotech, Les Ulis, France) and 1.6 unit of AMV reverse transcriptase (Promega, Madison, WI, USA) and then used as template for standard PCR. Standard amplification was carried out using Taq DNA polymerase (Invitrogen) in 25 µl final volume with the cycling conditions set at 94°C for 5 min followed by 35 cycles of 94°C for 1 min, 62°C for 1 min and 72°C for 1 min and ending by an elongation at 72°C for 7 min. Primers amplified a 259 and 113 bp fragment of iNOS and GAPDH cDNA, respectively. Primers used were: iNOS sense 5′-CGGTGCTGTATTTCCTTACGAGGCGAAGAAGG-3′, iNOS reverse 5′-GGTGCTGTCTGTTAGGAGGTCAAGTAAAGGGC-3′; GAPDH sense 5′-GTGAAGGTCGGAGTCAACG-3′, GAPDH reverse 5′-TGAGGTCAATGAAGGGGTC-3′.

### Blocking experiments

HaCaT cells were grown on two separate 96-well plates and pre-incubated with neutralizing anti-human TLR-2 mAb TLR-2.1 (10 µg/ml) (eBioscience, San Diego, California) and anti-human CD36 monoclonal antibody FA6-152 (Hycult biotechnology b.v) or isotype-matched control-purified mouse IgG antibodies (10 µg/ml) (Caltag) (10 µg/ml) diluted in supplemented DMEM media for IL-8 measurement, and in sterile PBS pH 7.4 for O_2_^•−^ quantitation at 37°C in 5% CO_2_ atmosphere. After 2 h, cells were incubated for 30 min with 100 µl DHE at 5 µM final concentration. After three washes, cells were incubated with 100 µl of a suspension of *P. acnes* (Abs at 600 nm = 1.0) in PBS and fluorescence intensity was recorded every 30 min over the 3 h time-frame stimulation. After 3 h of incubation, supernatants were collected and used for IL-8 quantitation as described below.

### Generation of O_2_^•−^ solution

A 10 mM O_2_^•−^ solution was obtained by mixing 16 mM dicylohexano-18-crown-6 with 9.8 mM KO_2_ in DMSO. The solution was allowed to stabilize for 1 h at room temperature with stirring and protected from light before use. The relative sensitivity of HaCaT and of *P. acnes* was then tested against serial dilution of the O_2_^•−^ solution.

### Statistical analysis

The statistical significance of differences between data from experimental groups was analyzed by paired Student's-test. A level of *P*≤0.05 was accepted as significant. Statistical significance is indicated by * (P≤0.05), ** (P≤0.01), and *** (P≤0.001), respectively.

### Accession numbers of genes and proteins

Catalase (# P04040), CD-36 (# P16671), ERK (# P28482), GpX (# P07203), GM-CSF (# P32927), IL-1α (# P01583), IL-1β (# P01584), IL-8 (# P10145), iNOS (# P35228), MnSOD (# Q7Z7M6), NOX1 (# Q9Y5S8), p38 (# Q16539), TLR2 (# O60603), TLR4 (# O00206), TLR6 (# Q9Y2C9), TNF-α (# P01375).

## Supporting Information

Figure S1 Effect of anti-acne treatments on the cell viability. HaCaT cells were untreated (black bar) or incubated for 18 h with *P. acnes* alone (MOI of 50) (gray bar) or with *P. acnes* in the presence of nicotinamide, zinc sulfate, doxyciclyne, nitroimidazole, retinol, retinoic acid, and isotretinoin at 0.01% (dark gray bar) or 0.05% (white bar). Cell viability was estimated by the cristal violet assay as described in Materials and Methods. Data are means±SD of two separate experiments.
(1.13 MB TIF)

Figure S2 Toxicity of anti-acne treatments on the keratinocytes. HaCaT cells were untreated (black bar) or incubated for 18 h with *P. acnes* alone (MOI of 50) (gray bar) or in the presence of nicotinamide, zinc sulfate, doxyciclyne, nitroimidazole, retinol, retinoic acid, or isotretinoin alone at 0.01% (dark gray bar) or 0.05% (white bar). Cell death was estimated spectrofluorometrically using YO-PRO-1 as described in Materials and Methods. Data are means±SD of two separate experiments.
(1.36 MB DOC)

Figure S3 Detection of Nox1 expression level in HaCaT treated with Nox1A-siRNA. HaCaT cells were pretreated with Nox1A-siRNA sequence against Nox1 of human NADPH oxidase or with a scrambled sequence used as a negative control as described in Materials and Methods. Cells were lysed in the buffer containing 250 mM Tris-HCl pH 6.8, 150 mM NaCl, 4% (W∶V) SDS, 0.5 mM EGTA, 5 mM DTT, 20 µg/ml leupeptin, 10 µg/ml aprotinin, 2 mM PMSF. Proteins were separated by 12.5% SDS-PAGE and transferred onto nitrocellulose membrane. The membrane was saturated with 5% non-fat milk in 0.1% Tween 20 - PBS for 1 h at room temperature and then incubated for 18 h at 4°C with primary polyclonal antibody against Nox1 (Santa Cruz Biotechnology Inc., Santa Cruz, CA) diluted at 2 µg/ml in 2.5% non-fat milk in PBS. Bound antibodies were detected by the conjugate anti-rabbit IgG-HRP and visualized using enhanced chemiluminescence system. β-actin levels are showed as loading controls.
(3.05 MB TIF)

## References

[1] Ingham E (1999). The immunology of *Propionibacterium acnes* and acne.. Curr Opin Infect Dis.

[2] Vowels BR, Yang S, Leyden JJ (1995). Induction of proinflammatory cytokines by a soluble factor of *Propionibacterium acnes*: implications for chronic inflammatory acne.. Infect Immun.

[3] Nagy I, Pivarcsi A, Koreck A, Szell M, Urban E (2005). Distinct strains of *Propionibacterium acnes* induce selective human beta-defensin-2 and interleukin-8 expression in human keratinocytes through toll-like receptors.. J Invest Dermatol.

[4] Graham GM, Farrar MD, Cruse-Sawyer JE, Holland KT, Ingham E (2004). Proinflammatory cytokine production by human keratinocytes stimulated with *Propionibacterium acnes* and *P. acnes* GroEL.. Br J Dermatol.

[5] Schaller M, Loewenstein M, Borelli C, Jacob K, Vogeser M (2005). Induction of a chemoattractive proinflammatory cytokine response after stimulation of keratinocytes with *Propionibacterium acnes* and coproporphyrin III.. Br J Dermatol.

[6] Yang CS, Shin DM, Lee HM, Son JW, Lee SJ (2008). ASK1-p38 MAPK-p47phox activation is essential for inflammatory responses during tuberculosis via TLR2-ROS signalling.. Cell Microbiol.

[7] Mahe YF, Martin R, Aubert L, Billoni N, Collin C (2006). Induction of the skin endogenous protective mitochondrial MnSOD by *Vitreoscilla filiformis* extract.. Int J Cosmet Sci.

[8] Ding W, Hudson LG, Liu KJ (2005). Inorganic arsenic compounds cause oxidative damage to DNA and protein by inducing ROS and RNS generation in human keratinocytes.. Mol Cell Biochem.

[9] Chang H, Oehrl W, Elsner P, Thiele JJ (2003). The role of H_2_O_2_ as a mediator of UVB-induced apoptosis in keratinocytes.. Free Radic Res.

[10] Aitken GR, Henderson JR, Chang SC, McNeil CJ, Birch-Machin MA (2007). Direct monitoring of UV-induced free radical generation in HaCaT keratinocytes.. Clin Exp Dermatol.

[11] Akamatsu H, Horio T, Hattori K (2003). Increased hydrogen peroxide generation by neutrophils from patients with acne inflammation.. Int J Dermatol.

[12] Kurutas EB, Arican O, Sasmaz S (2005). Superoxide dismutase and myeloperoxidase activities in polymorphonuclear leukocytes in acne vulgaris.. Acta Dermatovenerol Alp Panonica Adriat.

[13] Abdel Fattah NS, Shaheen MA, Ebrahim AA, El Okda ES (2008). Tissue and blood superoxide dismutase activities and malondialdehyde levels in different clinical severities of acne vulgaris.. Br J Dermatol.

[14] Fridovich I (1999). Fundamental aspects of reactive oxygen species, or what's the matter with oxygen?. Ann N Y Acad Sci.

[15] Valencia A, Kochevar IE (2008). Nox1-based NADPH oxidase is the major source of UVA-induced reactive oxygen species in human keratinocytes.. J Invest Dermatol.

[16] Rezvani HR, Mazurier F, Cario-Andre M, Pain C, Ged C (2006). Protective effects of catalase overexpression on UVB-induced apoptosis in normal human keratinocytes.. J Biol Chem.

[17] Crow JP, Ischiropoulos H (1996). Detection and quantitation of nitrotyrosine residues in proteins: *in vivo* marker of peroxynitrite.. Methods Enzymol.

[18] Deliconstantinos G, Villiotou V, Stavrides JC (1996). Increase of particulate nitric oxide synthase activity and peroxynitrite synthesis in UVB-irradiated keratinocyte membranes.. Biochem J.

[19] Pivarcsi A, Bodai L, Rethi B, Kenderessy-Szabo A, Koreck A (2003). Expression and function of Toll-like receptors 2 and 4 in human keratinocytes.. Int Immunol.

[20] Kollisch G, Kalali BN, Voelcker V, Wallich R, Behrendt H (2005). Various members of the Toll-like receptor family contribute to the innate immune response of human epidermal keratinocytes.. Immunology.

[21] Jugeau S, Tenaud I, Knol AC, Jarrousse V, Quereux G (2005). Induction of toll-like receptors by *Propionibacterium acnes*.. Br J Dermatol.

[22] Kim J, Ochoa MT, Krutzik SR, Takeuchi O, Uematsu S (2002). Activation of toll-like receptor 2 in acne triggers inflammatory cytokine responses.. J Immunol.

[23] Trivedi NR, Gilliland KL, Zhao W, Liu W, Thiboutot DM (2006). Gene array expression profiling in acne lesions reveals marked upregulation of genes involved in inflammation and matrix remodeling.. J Invest Dermatol.

[24] Schmuth M, Ortegon AM, Mao-Qiang M, Elias PM, Feingold KR (2005). Differential expression of fatty acid transport proteins in epidermis and skin appendages.. J Invest Dermatol.

[25] Nishimura S, Akagi M, Yoshida K, Hayakawa S, Sawamura T (2004). Oxidized low-density lipoprotein (ox-LDL) binding to lectin-like ox-LDL receptor-1 (LOX-1) in cultured bovine articular chondrocytes increases production of intracellular reactive oxygen species (ROS) resulting in the activation of NF-kappaB.. Osteoarthritis Cartilage.

[26] Cho S, Park EM, Febbraio M, Anrather J, Park L (2005). The class B scavenger receptor CD36 mediates free radical production and tissue injury in cerebral ischemia.. J Neurosci.

[27] Triantafilou M, Gamper FG, Haston RM, Mouratis MA, Morath S (2006). Membrane sorting of toll-like receptor (TLR)-2/6 and TLR2/1 heterodimers at the cell surface determines heterotypic associations with CD36 and intracellular targeting.. J Biol Chem.

[28] Nagaoka M, Kamisango K, Fujii H, Uchikawa K, Sekikawa I (1985). Structure of acidic polysaccharide from cell wall of *Propionibacterium acnes* strain C7.. J Biochem.

[29] Whale GA, Sutcliffe IC, Morrisson AR, Pretswell EL, Emmison N (2004). Purification and characterisation of lipoglycan macroamphiphiles from *Propionibacterium acnes*.. Antonie Van Leeuwenhoek.

[30] Lambeth JD, Kawahara T, Diebold B (2007). Regulation of Nox and Duox enzymatic activity and expression.. Free Radic Biol Med.

[31] Chamulitrat W, Schmidt R, Tomakidi P, Stremmel W, Chunglok W (2003). Association of gp91phox homolog Nox1 with anchorage-independent growth and MAP kinase-activation of transformed human keratinocytes.. Oncogene.

[32] Kang YJ, Seit-Nebi A, Davis RJ, Han J (2006). Multiple activation mechanisms of p38alpha mitogen-activated protein kinase.. J Biol Chem.

[33] Ikeda Y, Murakami A, Fujimura Y, Tachibana H, Yamada K (2007). Aggregated ursolic acid, a natural triterpenoid, induces IL-1beta release from murine peritoneal macrophages: role of CD36.. J Immunol.

[34] Kiningham KK, Cardozo ZA, Cook C, Cole MP, Stewart JC (2008). All-trans-retinoic acid induces manganese superoxide dismutase in human neuroblastoma through NF-kappaB.. Free Radic Biol Med.

[35] Peterson GL (1983). Determination of total protein.. Methods Enzymol.

[36] Boukamp P, Petrussevska RT, Breitkreutz D, Hornung J, Markham A (1988). Normal keratinization in a spontaneously immortalized aneuploid human keratinocyte cell line.. J Cell Biol.

[37] Laurent A, Nicco C, Chereau C, Goulvestre C, Alexandre J (2005). Controlling tumor growth by modulating endogenous production of reactive oxygen species.. Cancer Res.

[38] Alexandre J, Nicco C, Chereau C, Laurent A, Weill B (2006). Improvement of the therapeutic index of anticancer drugs by the superoxide dismutase mimic mangafodipir.. J Natl Cancer Inst.

[39] Servettaz A, Guilpain P, Goulvestre C, Chereau C, Hercend C (2007). Radical oxygen species production induced by advanced oxidation protein products predicts clinical evolution and response to treatment in systemic sclerosis.. Ann Rheum Dis.

