# Supplementary figures and images for: Production of Superoxide Anions by Keratinocytes Initiates P. acnes-Induced Inflammation of the Skin
Source: PLoS Pathog. 2009 Jul 24;5(7):e1000527. doi: 10.1371/journal.ppat.1000527 (PMC2709429; doi:10.1371/journal.ppat.1000527)

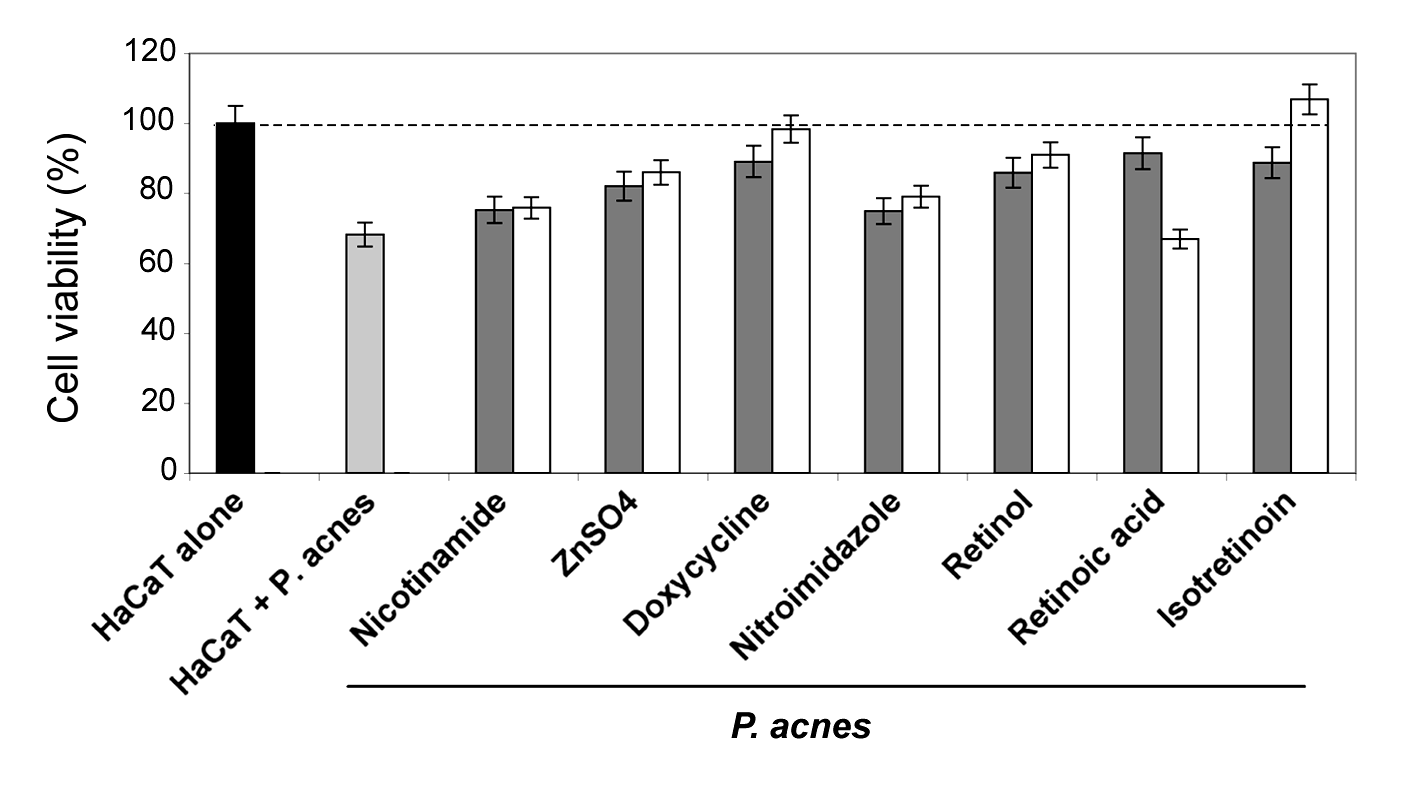

Supplement: Figure S1 — Effect of anti-acne treatments on the cell viability. HaCaT cells were untreated (black bar) or incubated for 18 h with P. acnes alone (MOI of 50) (gray bar) or with P. acnes in the presence of nicotinamide, zinc sulfate, doxyciclyne, nitroimidazole, retinol, retinoic acid, and isotretinoin at 0.01% (dark gray bar) or 0.05% (white bar). Cell viability was estimated by the cristal violet assay as described in Materials and Methods. Data are means±SD of two separate experiments. (1.13 MB TIF) [file ppat.1000527.s001.tif]

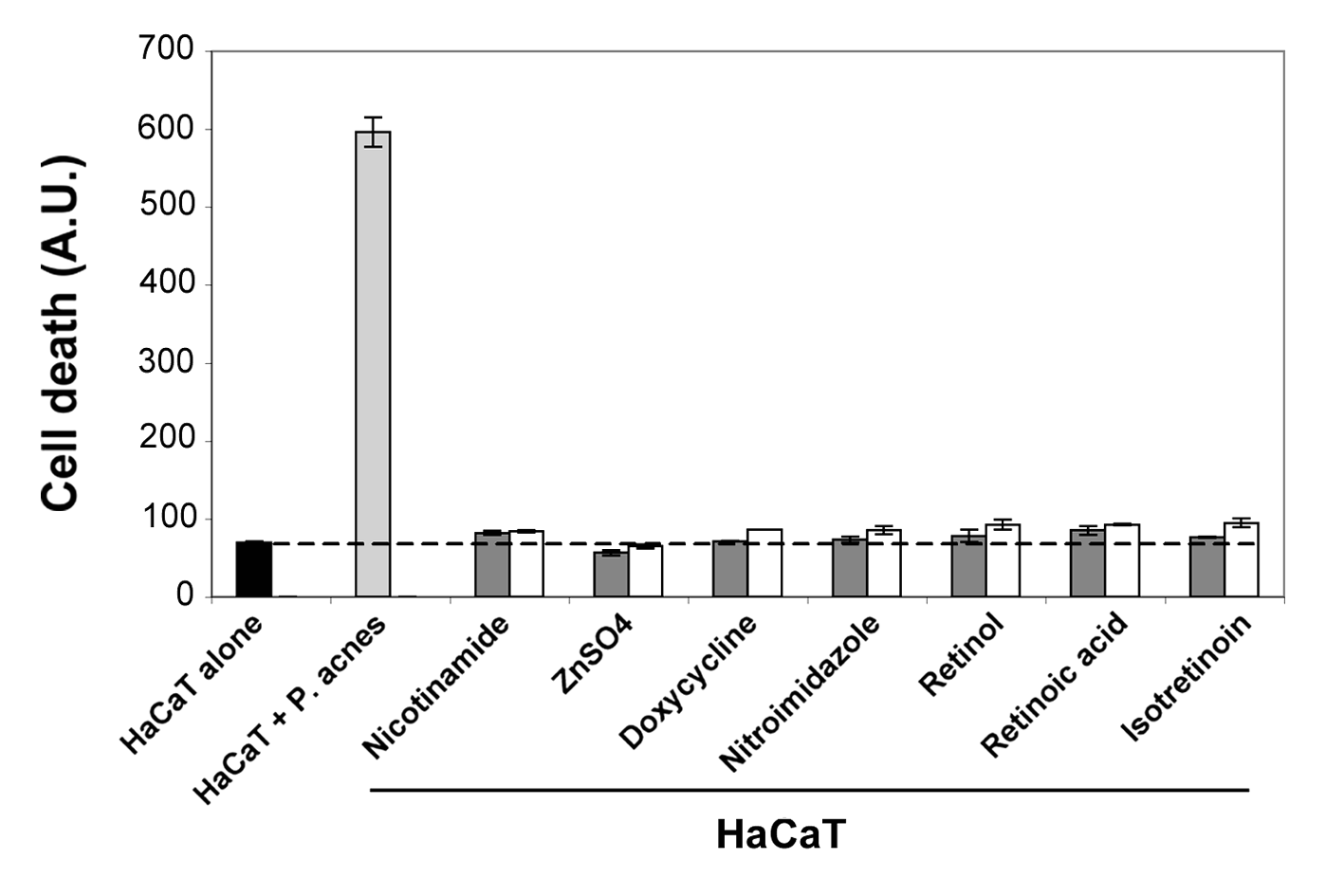

Supplement: Figure S2 — Toxicity of anti-acne treatments on the keratinocytes. HaCaT cells were untreated (black bar) or incubated for 18 h with P. acnes alone (MOI of 50) (gray bar) or in the presence of nicotinamide, zinc sulfate, doxyciclyne, nitroimidazole, retinol, retinoic acid, or isotretinoin alone at 0.01% (dark gray bar) or 0.05% (white bar). Cell death was estimated spectrofluorometrically using YO-PRO-1 as described in Materials and Methods. Data are means±SD of two separate experiments. (1.36 MB DOC) [file ppat.1000527.s002.tif]

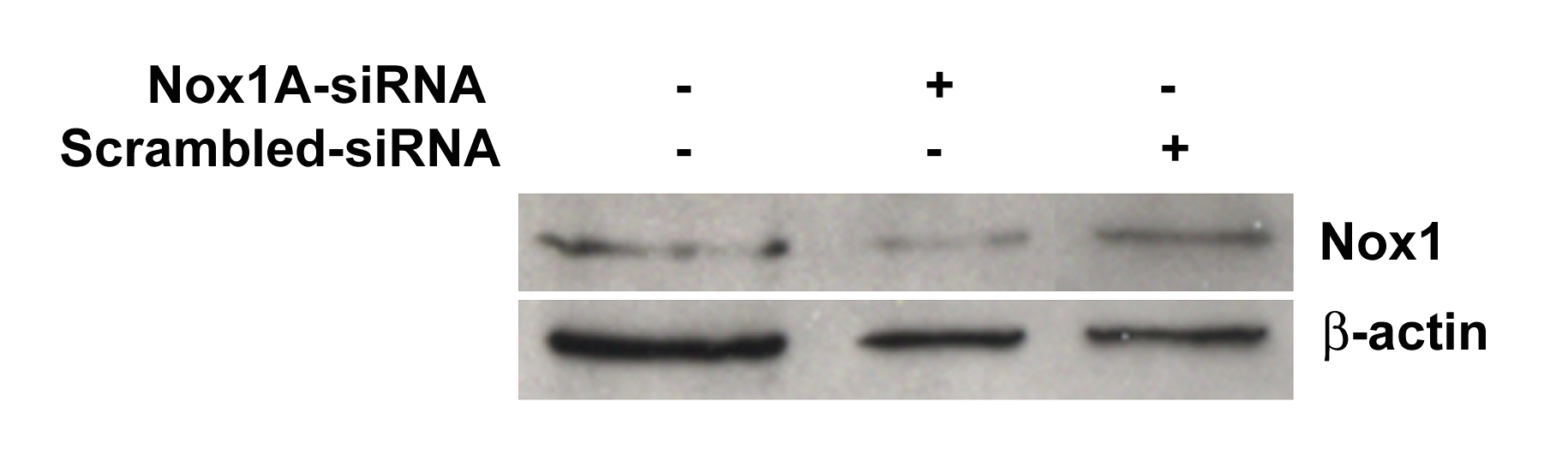

Supplement: Figure S3 — Detection of Nox1 expression level in HaCaT treated with Nox1A-siRNA. HaCaT cells were pretreated with Nox1A-siRNA sequence against Nox1 of human NADPH oxidase or with a scrambled sequence used as a negative control as described in Materials and Methods. Cells were lysed in the buffer containing 250 mM Tris-HCl pH 6.8, 150 mM NaCl, 4% (W∶V) SDS, 0.5 mM EGTA, 5 mM DTT, 20 µg/ml leupeptin, 10 µg/ml aprotinin, 2 mM PMSF. Proteins were separated by 12.5% SDS-PAGE and transferred onto nitrocellulose membrane. The membrane was saturated with 5% non-fat milk in 0.1% Tween 20 - PBS for 1 h at room temperature and then incubated for 18 h at 4°C with primary polyclonal antibody against Nox1 (Santa Cruz Biotechnology Inc., Santa Cruz, CA) diluted at 2 µg/ml in 2.5% non-fat milk in PBS. Bound antibodies were detected by the conjugate anti-rabbit IgG-HRP and visualized using enhanced chemiluminescence system. β-actin levels are showed as loading controls. (3.05 MB TIF) [file ppat.1000527.s003.tif]
